# Identification and validation of an interpretable EEG-based machine learning model for the diagnosis of post-stroke cognitive impairment

**DOI:** 10.3389/fnagi.2025.1700771

**Published:** 2026-01-12

**Authors:** Xinyang Wang, Jian Song, Weicheng Kong, Wei Wei, Haoran Shi, Peitao Xu, Yuqing Zhao, Jiayu Cai, Xiehua Xue

**Affiliations:** 1 The Affiliated Rehabilitation Hospital, Fujian University of Traditional Chinese Medicine, Fuzhou, China; 2 College of Rehabilitation Medicine, Fujian University of Traditional Chinese Medicine, Fuzhou, China; 3 Fujian Provincial Key Laboratory of Rehabilitation Technology, Fujian Key Laboratory of Cognitive Rehabilitation, Fujian Provincial Rehabilitation Industrial Institution, Fuzhou, China

**Keywords:** electroencephalography, machinelearning, post-stroke cognitive impairment, prediction mode, SHAP, stroke

## Abstract

**Introduction:**

Post-stroke cognitive impairment (PSCI) is a prevalent and disabling consequence of stroke, yet objective tools for its early identification are lacking. This study aimed to develop and validate an interpretable machine learning (ML) model based on electroencephalography (EEG) to support the early detection of PSCI.

**Methods:**

We conducted a study involving 174 participants, including stroke patients with and without cognitive impairment and age-matched healthy controls. Resting-state EEG was acquired from all subjects, and multidimensional features, including power spectral ratios and microstate parameters, were extracted. Feature selection was performed using LASSO regression, random forest, and the Boruta algorithm. Five machine learning models were evaluated and compared based on their area under the curve (AUC), accuracy, Brier score, calibration plots, and decision curve analysis. Model interpretability was explained using SHAP (Shapley Additive Explanations). The final validated model was deployed as an interactive web-based application.

**Results:**

Seven EEG features were identified as most predictive of PSCI: the delta-plus-theta to alpha-plus-beta ratio (DTABR) in frontal, central, and global regions; the mean microstate duration of classes A and B (A-MMD, B-MMD); the mean frequency of microstate D (D-MFO); and the mean coverage of microstate A (A-MC). The random forest model demonstrated the highest performance (AUC = 0.91, accuracy = 0.83, specificity = 0.88, Brier score = 0.12), alongside satisfactory calibration and a positive net clinical benefit. The model was further validated on an independent external cohort (*n* = 42), showing robust predictive performance (AUC = 0.97, accuracy = 0.90). An accessible web tool was created for individualized risk prediction (https://eeg-predict.streamlit.app/).

**Discussion:**

The findings suggest that an interpretable EEG-based ML model can provide accurate early screening of PSCI. Integration of this approach into clinical workflows may support personalized rehabilitation strategies and optimize post-stroke care. Future studies are warranted to validate the model in larger, multicenter cohorts.

## 1 Introduction

Stroke remains one of the leading causes of long-term disability and mortality worldwide, with a substantial proportion of survivors developing post-stroke cognitive impairment (PSCI), a common and serious complication affecting approximately 44–59% of stroke patients (Aam et al., 2020; Lo et al., 2019). PSCI not only impairs memory, attention, and executive function but also reduces functional independence, increases the risk of recurrent stroke, and worsens long-term prognosis (El Husseini et al., 2023). With the increasing global burden of stroke, especially in aging populations, early detection and intervention of PSCI have become a critical clinical priority (El Husseini et al., 2023).

Computed tomography (CT) and magnetic resonance imaging (MRI) are commonly used to screen for high-risk PSCI cases, but CT lacks standardized diagnostic thresholds (Ball et al., 2022, 2023). Although MRI provides valuable structural insights, it still requires cognitive assessments for diagnostic confirmation (Ball et al., 2023) and it remains costly and less accessible (Bhat et al., 2021). The Montreal Cognitive Assessment (MoCA) is widely used for this purpose due to its high sensitivity and specificity in detecting PSCI (Wei et al., 2025). However, cognitive tests can depend on patient cooperation, which may limit consistency (Pasi et al., 2013; Yan et al., 2022).

Given the limitations of current structural imaging and cognitive screening methods, there is an increasing need for objective neurophysiological tools that can directly reflect functional brain disturbances. Electroencephalography (EEG), as a noninvasive, accessible, and cost-effective technique, provides high temporal resolution for capturing real-time neural activity (Hadiyoso et al., 2023; Tarailis et al., 2023). EEG power spectral density and microstate features have shown promise in capturing cognitive dysfunction in PSCI. Prior studies report that patients with PSCI exhibit distinct alterations in power ratios—such as the Delta/Alpha Ratio (DAR), Delta/Theta Ratio (DTR), and (Delta + Theta)/(Alpha + Beta) Ratio (DTABR)—which serve as potential biomarkers for diagnosis and prognosis (Hussain et al., 2021; Tarailis et al., 2023). In addition to spectral power, EEG microstates—dynamic brain activity patterns—have also been studied in PSCI. Likewise, microstate parameters (A–D), including mean duration (MMD), frequency of occurrence (MFO), and coverage (MC), are suggested as supplementary indicators of stroke-related cognitive impairment (Hao et al., 2022).

Since PSCI involves complex brain changes, combining EEG markers improves detection. This makes machine learning ideal for analysis—recent studies show ML effectively predicts post-stroke cognitive outcomes by integrating these EEG features. For instance, Lee et al. (2023a,b) demonstrated that brain network attributes extracted from acute-phase EEG can predict MoCA scores at 3 months post-stroke (Lee et al., 2023a,b). In addition, interpretability tools such as SHAP (Shapley Additive Explanations) help visualize how individual features influence predictions, enhancing clinical trust and facilitating practical decision-making. These developments support the integration of interpretable ML and EEG for early, accessible, and personalized assessment of PSCI risk (Nohara et al., 2022).

Despite the recognized value of EEG as a non-invasive and cost-effective tool for brain monitoring, current PSCI prediction models remain limited in scope, interpretability, and methodological rigor. While machine learning applications in stroke medicine are expanding, recent systematic reviews indicate that they predominantly focus on general stroke diagnosis (Daidone et al., 2024) or functional disability related to motor recovery (Sood et al., 2024), rather than specifically targeting the complex mechanisms of PSCI. Furthermore, many studies rely on isolated EEG features—typically spectral power or coherence—which fail to capture the multidimensional dynamics of post-stroke brain activity. Hadiyoso et al. and Asadi et al. have noted the methodological heterogeneity and the underutilization of functional connectivity features in existing research (Asadi et al., 2023; Hadiyoso et al., 2023). Critically, relying solely on spectral features neglects the dynamic temporal organization of brain networks. Recent evidence by Hao et al. (2022) suggests that EEG microstates reflect global network stability and compensatory reconfiguration following stroke, capturing neural disturbances that spectral power alone may miss.

Another major limitation in prior PSCI studies is the lack of specificity in control group selection; comparisons often exclude stroke patients without cognitive impairment (PSN), making it difficult to determine whether observed EEG alterations are driven by cognitive dysfunction or by stroke pathology itself. Moreover, although some recent studies have applied machine learning and SHAP visualization to improve interpretability, these approaches typically rely on single-domain EEG features, lack external validation, and offer limited clinical applicability.

These gaps underscore the need for a comprehensive, explainable, and clinically grounded model. Unlike previous studies, the present work advances PSCI research by integrating multidimensional EEG markers (frequency-domain power ratios and time-domain microstate dynamics) within an interpretable machine learning framework and validating the model across an independent dataset with both PSN and HC controls—thereby enhancing both feature specificity and clinical usability.

To address these limitations and bridge the gap between high-dimensional feature fusion and clinical precision, this study aimed to identify and validate an interpretable ML-based diagnostic model for PSCI by combining multiple EEG domains within a unified framework. By incorporating both PSN and HC as non-impaired reference groups, we ensured that the extracted EEG biomarkers specifically reflected cognitive dysfunction rather than general stroke-related abnormalities. Leveraging SHAP for model transparency, the final classifier was further deployed as a user-friendly web-based tool to facilitate early risk stratification in clinical practice. The goal is to provide an efficient, objective, and clinically applicable solution to support early detection and personalized management of PSCI.

## 2 Materials and methods

### 2.1 Study design and participants

This study was approved by the Ethics Committee of the Affiliated Rehabilitation Hospital of Fujian University of Traditional Chinese Medicine (Approval No: 2024YJS-003-01), and written informed consent was obtained from all participants prior to enrollment. Participants were recruited from the Department of Neurology at The Affiliated Rehabilitation Hospital of Fujian University of Traditional Chinese Medicine. The main dataset (training and test sets) was collected between October 2024 and June 2025, and an independent external validation cohort was prospectively collected during the subsequent three-month period (July–September 2025) to assess model generalizability.

Based on cognitive status and history of stroke, they were categorized into three groups: post-stroke cognitive impairment (PSCI group), post-stroke non-cognitive impairment group (PSN group), and healthy controls group (HC group). Inclusion criteria for the PSCI and PSN groups included age 40–75 years, first-ever ischemic or hemorrhagic stroke within 6 months, and the ability to comply with cognitive assessments. For the PSCI group, specific inclusion criteria included (1) a clinical diagnosis of cognitive impairment confirmed by a neurologist based on clinical presentation and cognitive evaluation, and (2) a Montreal Cognitive Assessment (MoCA) score below the established cutoff (<25). For the PSN group, specific inclusion criteria included (1) no clinical diagnosis of cognitive impairment by the attending neurologist, and (2) a MoCA score within the normal range (≥25) (Potocnik et al., 2020). HC participants were aged 40–80 years, had no history of stroke, neurological, or psychiatric disorders, and scored ≥25 on the MoCA. Exclusion criteria included cognitive dysfunction from non-stroke causes, prior stroke with sequelae, impaired consciousness, sensory or speech disabilities, fever or unstable vitals, and organ failure.

### 2.2 Sample size calculation

The sample size was determined based on the events per variable (EPV) principle, a commonly applied approach in predictive modeling and regression analysis (van Smeden et al., 2019; Vittinghoff and McCulloch, 2007). Considering the reported incidence of cognitive impairment (CI) at 3 months post-stroke is approximately 0.59 (Aam et al., 2020), and aiming to include seven predictor variables with an EPV threshold of 10, the minimum required sample size was estimated using the following formula:

$\text{Sample size}=\frac{\text{Number of variables}\times \mathrm{EPV}}{1-\text{Incidence rate}}=\frac{7\times 10}{1-0.59}=171$

### 2.3 Determination of participants

The classification of participants was based on their clinical history of stroke and cognitive performance. Stroke history was verified through the hospital’s electronic medical records at the Rehabilitation Hospital of Fujian University of Traditional Chinese Medicine and confirmed by directly interviewing each participant or their legal representative.

Cognitive function was assessed using the Montreal Cognitive Assessment (MoCA), a widely validated screening tool for detecting cognitive impairment. The total MoCA score ranges from 0 to 30, with lower scores indicating poorer cognitive performance. In this study, a MoCA score of <25 was used as the threshold to define cognitive impairment, consistent with prior research in PSCI populations (Potocnik et al., 2020). All MoCA assessments were conducted by trained clinicians who were blinded to EEG results to reduce assessment bias.

### 2.4 Data collection

Basic demographic information, including sex and age, was collected from all participants. Resting-state EEG signals were recorded from 19 scalp electrodes based on the international 10–20 system for patients with PSCI, PSN, and HC. A total of 24 EEG-derived features were extracted for analysis. These included three power ratio indices—delta/alpha ratio (DAR), delta/theta ratio (DTR), and (delta + theta)/(alpha + beta) ratio (DTABR)—calculated across four anatomical regions (global, frontal, central, and posterior). In addition, microstate analysis was performed to extract three temporal parameters for each of the four canonical EEG microstates (A–D): (1) Mean Median Duration (MMD), defined as the average duration of each microstate occurrence, reflecting the temporal stability of a brain network; (2) Mean Frequency of Occurrence (MFO), defined as the number of times a microstate occurs per second, indicating the activation rate of a network; and (3) Mean Coverage (MC), defined as the proportion of total analysis time covered by a given microstate, representing its overall dominance.

### 2.5 EEG signal processing and feature extraction method

#### 2.5.1 Acquiring and preprocessing EEG signals

**Figure 1:**
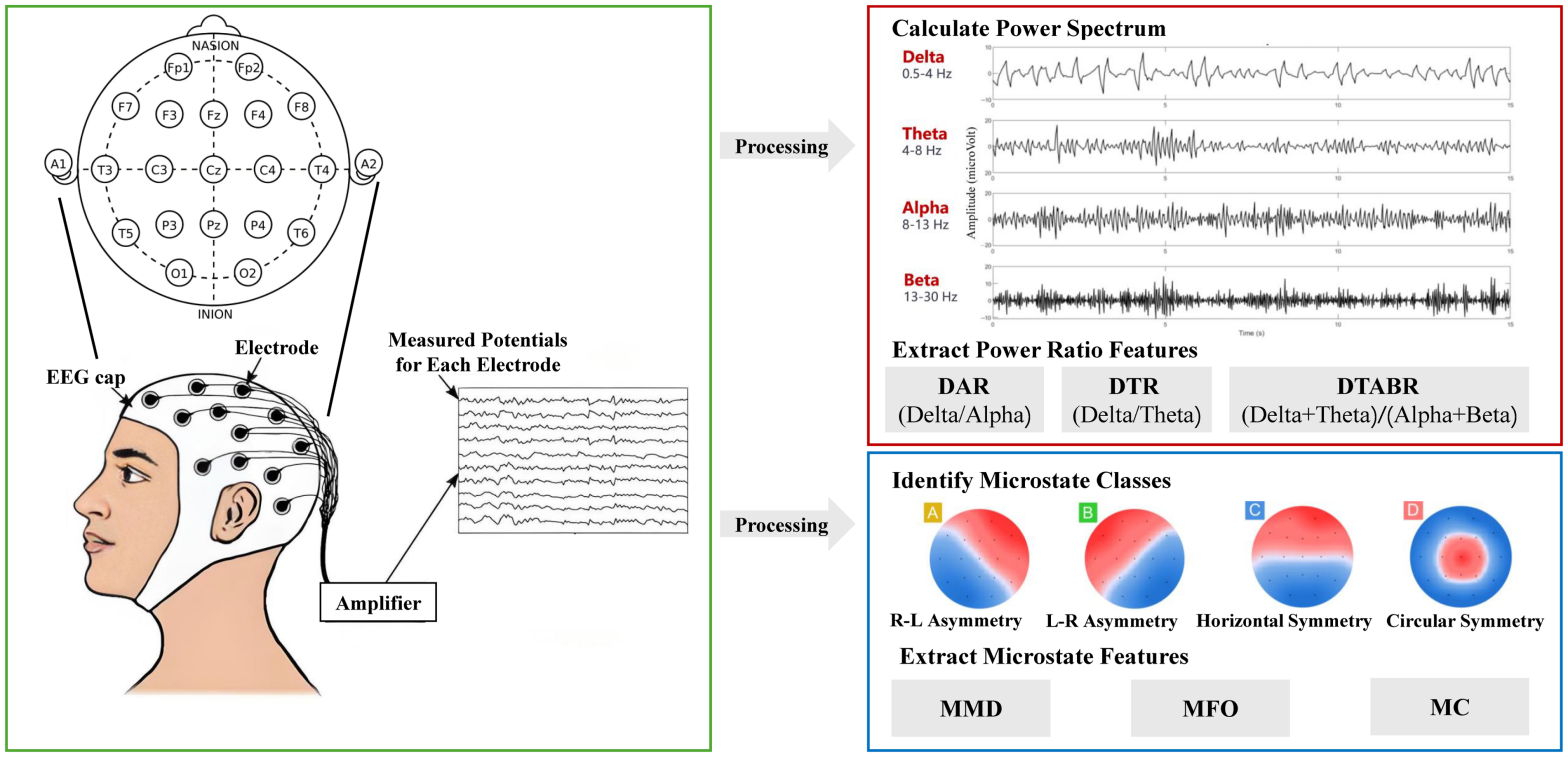
EEG signal processing and feature extraction method. This diagram illustrates the workflow for preprocessing and analyzing EEG signals. Raw EEG data were collected and preprocessed. Spectral power features were extracted, and key power ratios were computed, including DAR (Delta/Alpha Ratio), DTR (Delta/Theta Ratio), and DTABR (Delta + Theta/Alpha + Beta Ratio). In parallel, EEG microstate analysis identified four canonical microstates (A–D), from which temporal parameters were derived: Mean Median Duration (MMD), Mean Frequency of Occurrence (MFO), and Mean Coverage (MC).

As illustrated in Figure 1, continuous resting-state EEG signals were recorded at a sampling rate of 500 Hz for 3 min with participants’ eyes closed, using a 19-channel EEG cap arranged according to the international 10–20 system. EEG activity was captured by the NVX52 EEG acquisition system (model No. NVX52, Nanjing NeuroMed Technology Group Co., Ltd., China), an instrument designed for cognitive and autonomic function mapping. Electrode impedance was maintained below 10 kΩ and verified prior to each recording. All raw EEG data were saved in EDF format for offline processing.

Preprocessing was conducted in MATLAB R2019b using the EEGLAB toolbox (version 2021.1) and required approximately 5 min per participant. The workflow included visual inspection for data quality assessment, detection of nonfunctional or noisy channels followed by spherical spline interpolation, re-referencing to the common average reference, and artifact removal using Infomax independent component analysis (ICA). ICA components reflecting ocular, muscular, or movement-related artifacts were identified through topographical and temporal features and removed accordingly. Component rejection was based on both spatial and temporal characteristics (Delorme et al., 2007; von Wegner and Laufs, 2018). Recordings were excluded if more than 20% of data were corrupted or if more than three channels were nonfunctional, yielding an overall exclusion rate of 2.3%. All preprocessing and quality-control procedures were documented to ensure methodological reproducibility.

#### 2.5.2 Analyzing power ratio features from EEG signals

Following preprocessing, power spectral density (PSD) analysis was performed using Welch’s method via the pwelch function. A Hann window with a segment length of 4 s and 50% overlap was applied. PSD was calculated across a broadband range of 0.5–40 Hz, covering five standard EEG frequency bands: delta (0.5–4 Hz), theta (4–8 Hz), alpha (8–13 Hz), beta (13–30 Hz), and gamma (30–40 Hz).

For subsequent analysis, these power values were used to compute power ratio indices such as DAR (Delta/Alpha Ratio), DTR (Delta/Theta Ratio), and DTABR [(Delta + Theta)/(Alpha + Beta)] across four brain regions: global (all 19 electrodes), frontal, central, and posterior.

The analysis was conducted using EEGLAB functions implemented in MATLAB. Specifically, pop_select was used for regional channel selection, and MATLAB’s pwelch function was applied for spectral power analysis. All analyses were performed in MATLAB R2021a (The MathWorks, Inc.) with EEGLAB version 2021.1 on a Windows 10 64-bit workstation equipped with an Intel Core i7 processor and 32 GB RAM.

#### 2.5.3 Performing EEG microstate analysis

EEG microstate analysis was performed using the open-source Python toolbox developed by von Wegner and Laufs (2018). Preprocessed EEG data were band-pass filtered between 1 and 35 Hz. Global Field Power (GFP) was calculated on 60-s segments per participant, with a minimum peak spacing of 10 ms. GFP peaks exceeding two standard deviations from the mean were excluded to reduce outliers.

A modified K-means clustering algorithm with deterministic dynamic initialization was applied to extract four canonical microstates (A–D), based on predefined topographic patterns: (A) right–left asymmetry, (B) left–right asymmetry, (C) horizontal symmetry, and (D) circular symmetry (Musaeus et al., 2020; Pascual-Marqui et al., 1995). Each microstate class included a predefined number of candidate templates (7 for A, 5 for B, 6 for C, and 3 for D), resulting in 630 template combinations. These were iteratively evaluated (maximum 500 iterations, convergence threshold 10^−6^), and the configuration yielding the highest global explained variance (GEV) was selected. To minimize artifacts, segments shorter than 8 ms or longer than 120 ms were excluded.

For each microstate class, the three temporal parameters (MMD, MFO, MC) were computed to characterize the temporal dynamics of large-scale brain activity. All the analyses were conducted on the same workstation using Python 2.7.18. Standard scientific computing libraries were used, including NumPy (v1.16.6), SciPy (v1.2.3), matplotlib (v2.2.5), StatsModels (v0.10.2), and scikit-learn (v0.20.4). Band-pass filtering was implemented using a zero-phase 4th-order Butterworth filter via SciPy’s signal.filtfilt function. Clustering procedures employed polarity-invariant spatial correlation within the modified K-means framework, with all computations performed in double-precision floating-point format (float64). Statistical analyses were conducted using SciPy’s stats module and StatsModels for multiple-comparison correction. All analysis scripts were managed under Git version control (Git v2.23.0) to ensure complete reproducibility.

### 2.6 Data analysis

#### 2.6.1 Descriptive statistics and group comparisons

All statistical analyses were performed using GraphPad Prism (version 10.1.2) and Python (version 3.13). The normality of continuous variables was assessed using the Shapiro–Wilk test. Normally distributed variables were expressed as mean ± standard deviation (SD), and non-normally distributed variables were presented as median and interquartile range (IQR). Categorical variables were reported as counts.

For comparisons among the three groups (PSCI, PSN, and HC), one-way ANOVA was used for normally distributed variables, while the Kruskal–Wallis test was applied for non-normally distributed data. For two-group comparisons (Training set vs. Test set), the independent-samples *t*-test or Mann–Whitney *U* test was used depending on distribution. Chi-square test or Fisher’s exact test was used for categorical variables. A two-sided *p*-value < 0.05 was considered statistically significant.

#### 2.6.2 Feature processing and predictive modeling

The initial sample size estimation was guided by the events-per-variable (EPV) principle. With seven predictors and an EPV threshold of 10, the minimum required total sample size was 171. For model development and evaluation, the dataset was split into a training cohort (*n* = 121, containing 62 positive cases) and an independent test cohort (*n* = 52, containing 25 positive cases). The effective EPV in the training set was therefore 8.9. It is important to note that the machine learning algorithms employed are inherently regularized and have been demonstrated to be more robust in such lower-EPV scenarios than traditional unpenalized regression (Fusar-Poli et al., 2019).

Prior to model construction, the dataset was randomly split into a training set (70%) and a test set (30%) to prevent data leakage and ensure fair performance evaluation. Feature selection was performed using a combination of LASSO regression, Random Forest importance ranking, and the Boruta algorithm. Features identified in the intersection of these methods were retained as candidate predictors.

To develop the predictive models, all features were standardized prior to model construction. We implemented and compared five supervised machine learning classifiers: Random Forest (RF), Decision Tree (DT), Extreme Gradient Boosting (XGBoost), Support Vector Machine (SVM), and Logistic Regression (LR). The hyperparameters for all models were optimized using a repeated grid-search with 10-fold cross-validation on the training set to ensure robustness and prevent overfitting. For the Random Forest model, the optimal parameters were determined to be: random_state = 42, max_depth = None, min_samples_split = 10, and n_estimators = 50. The selection of a min_samples_split value greater than the default was a deliberate choice to increase the regularization of individual trees, thereby enhancing the model’s generalizability. Similarly, model-specific strategies such as regularization (for LR and SVM) and early stopping (for XGBoost) were employed to enhance generalization.

Model performance was assessed using metrics including Accuracy, AUC, Brier Score, Sensitivity, Specificity, Positive Predictive Value (PPV), Negative Predictive Value (NPV), and F1 Score. Discrimination and calibration were evaluated using ROC curves and calibration plots, while clinical usefulness was assessed via decision curve analysis (DCA).

#### 2.6.3 Interpretability and clinical implementation

To enhance model interpretability, Shapley Additive exPlanations (SHAP) were applied to quantify the impact and direction of each feature on prediction outcomes. SHAP plots visualized how individual EEG features influenced PSCI risk estimates, aiding in transparent and clinically meaningful interpretations.

For practical clinical application, the final Random Forest model was deployed using the Python-based Streamlit framework. The interactive web application allows input of patient-specific EEG features to return real-time PSCI risk predictions, accompanied by individualized SHAP force plots to support clinical decision-making. A clinical disclaimer for the web-based tool is provided in the Supplementary material 4.

## 3 Results

### 3.1 General characteristics

A total of 174 participants were included in the final analysis after excluding 12 individuals due to severe data loss (*n* = 4) or poor cooperation during EEG acquisition (*n* = 8). The dataset was randomly split into a training cohort (*n* = 121) and a test cohort (*n* = 53). The overall median age was 64 years, with 118 (67.81%) males and 56 (32.18%) females. Among all participants, 87 individuals were clinically diagnosed with PSCI.

**Figure 2:**
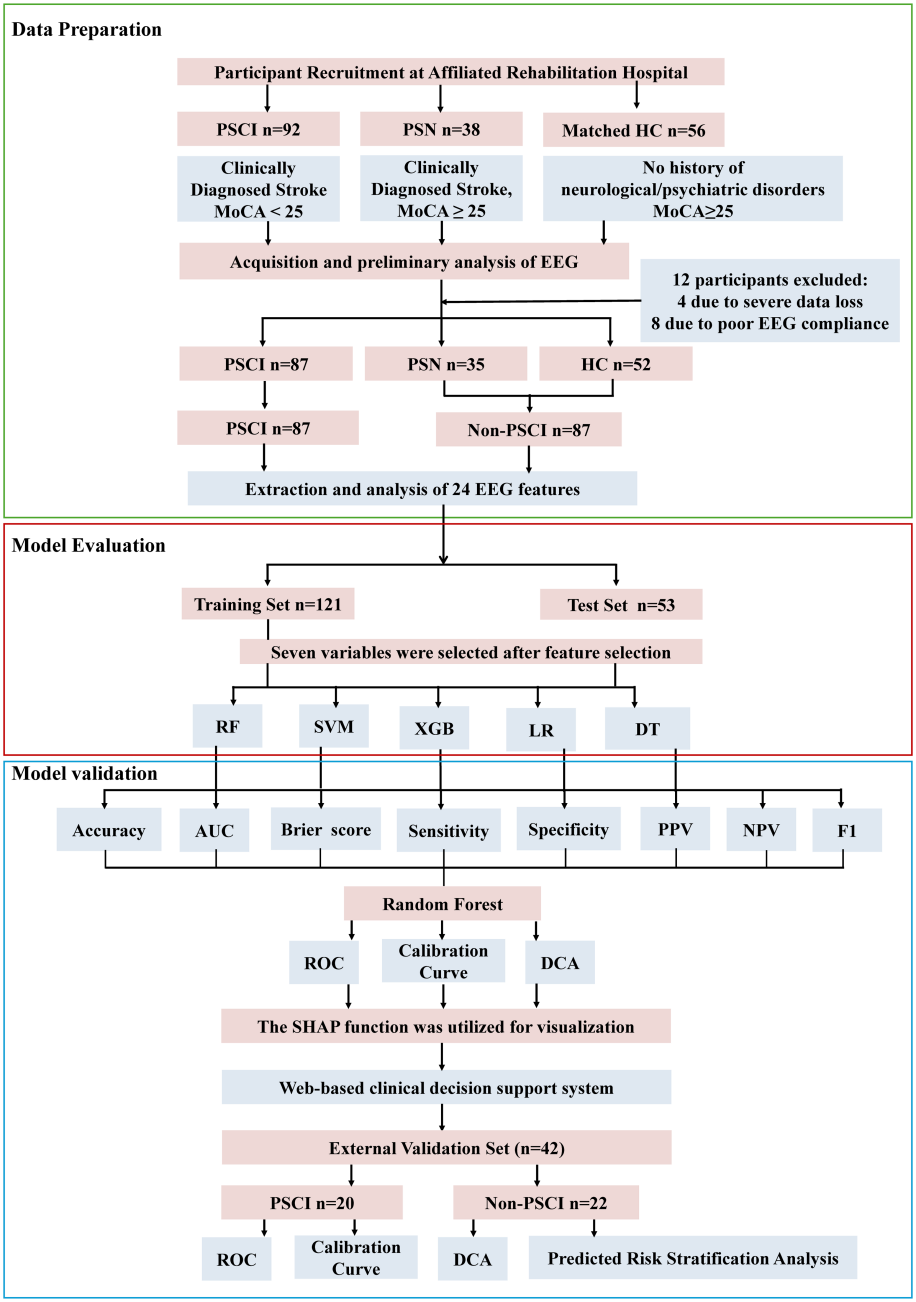
The flowchart of the study. Abbreviations in the flowchart include: PSCI (Post-Stroke Cognitive Impairment), PSN (Post-Stroke Non-Cognitive Impairment), HC (Healthy Control), MoCA (Montreal Cognitive Assessment), Non-PSCI (Non-Post-Stroke Cognitive Impairment; Combining PSN and HC), RF (Random Forest), SVM (Support Vector Machine), XGB (XGBoost), LR (Logistic Regression), DT (Decision Tree), ROC (Receiver Operating Characteristic Curve), DCA (Decision Curve Analysis), and SHAP (Shapley Additive Explanation).

Initial analyses revealed no significant differences in demographic or EEG characteristics between the post-stroke non-cognitive impairment (PSN) group and healthy controls (HC), with the exception of marginally significant variations in A-MMD (*p* = 0.01) and B-MMD (*p* = 0.03) (Supplementary material 1). Based on this overall comparability, we combined PSN and HC participants into a unified ‘non-PSCI’ group for subsequent analyses to improve statistical power and strengthen model robustness. Baseline comparisons between the training and test sets revealed no statistically significant differences across all key variables (all *p* > 0.05) except for gender, supporting the comparability of the two cohorts (Table 1). The participant enrollment flowchart is illustrated in Figure 2. To visualize the spatial distribution of spectral power, we present group-averaged topographies for the PSCI and non-PSCI groups across four frequency bands (delta, theta, alpha, beta) in Supplementary material 2.

**Table 1:** Baseline characteristics of participants in training and test set.

Characteristic	Total (*n* = 174)	Training set (*n* = 121)	Test set (*n* = 53)	*p*-value
Age	64.00 (55.00, 70.25)	63.00 (57.50, 71.00)	64.00 (53.00, 69.50)	0.35
Sex				<0.05
Male	95	80	15	
Female	79	41	38	
MoCA	24.50 (13.75, 26.00)	25.00 (14.00, 26.00)	24.00 (12.50, 26.00)	<0.001
DAR (global)	1.16 (0.56, 2.32)	1.26 (0.58, 2.46)	0.93 (0.53, 2.06)	0.48
DAR (frontal)	1.63 (0.74, 3.38)	1.84 (0.74, 3.47)	1.45 (0.75, 2.58)	0.36
DAR (central)	1.17 (0.60, 2.43)	1.20 (0.64, 2.51)	0.99 (0.57, 2.08)	0.38
DAR (posterior)	0.71 (0.32, 1.51)	0.77 (0.32, 1.56)	0.70 (0.31, 1.36)	0.77
DTR (global)	2.12 (1.37, 3.16)	2.29 (1.32, 3.26)	1.76 (1.39, 2.58)	0.13
DTR (frontal)	2.32 (1.53, 3.67)	2.54 (1.55, 3.88)	1.93 (1.50, 3.24)	0.13
DTR (central)	2.22 (1.43, 3.22)	2.36 (1.37, 3.47)	1.90 (1.54, 2.61)	0.14
DTR (posterior)	1.78 (1.56, 2.54)	1.96 (1.14, 2.68)	1.59 (1.17, 2.22)	0.17
DTABR (global)	1.23 (0.65, 2.25)	1.23 (0.66, 2.29)	1.23 (0.63, 2.10)	0.64
DTABR (frontal)	1.88 (0.88, 3.06)	2.00 (0.91, 3.08)	1.59 (0.85, 2.61)	0.59
DTABR (central)	1.26 (0.67, 2.41)	1.32 (0.68, 2.53)	1.13 (0.67, 2.08)	0.51
DTABR (posterior)	0.95 (0.42, 1.64)	0.94 (0.43, 1.68)	0.99 (0.39, 1.47)	0.88
A-MMD	45.09 (41.41, 49.60)	45.86 (41.37, 49.60)	44.27 (41.85, 49.39)	0.84
A-MFO	4.85 (4.19, 5.50)	4.86 (4.17, 5.45)	4.83 (4.25, 5.58)	0.91
A-MC	24.06 ± 5.12	24.38 ± 5.35	23.34 ± 4.54	0.22
B-MMD	45.08 (41.02, 49.54)	44.51 (41.35, 49.56)	45.88 (40.48, 49.60)	0.88
B-MFO	4.85 ± 0.99	4.88 ± 1.00	4.78 ± 0.97	0.55
B-MC	23.02 (20.54, 26.39)	23.17 (21.17, 26.98)	22.36 (19.34, 25.26)	0.29
C-MMD	48.35 ± 6.06	47.91 ± 6.27	49.35 ± 5.47	0.14
C-MFO	5.16 ± 1.17	5.09 ± 1.11	5.33 ± 1.30	0.22
C-MC	29.65 (24.50, 33.47)	29.02 (23.42, 33.00)	31.30 (25.67, 33.79)	0.05
D-MMD	42.77 (38.64, 46.52)	42.71 (38.61, 46.56)	43.47 (38.55, 46.70)	0.84
D-MFO	4.31 ± 1.06	4.35 ± 1.04	4.24 ± 1.11	0.55
D-MC	19.02 (16.17, 22.30)	19.64 (16.68, 22.40)	18.22 (15.38, 22.01)	0.23

### 3.2 Screening for predictive factors

**Figure 3:**
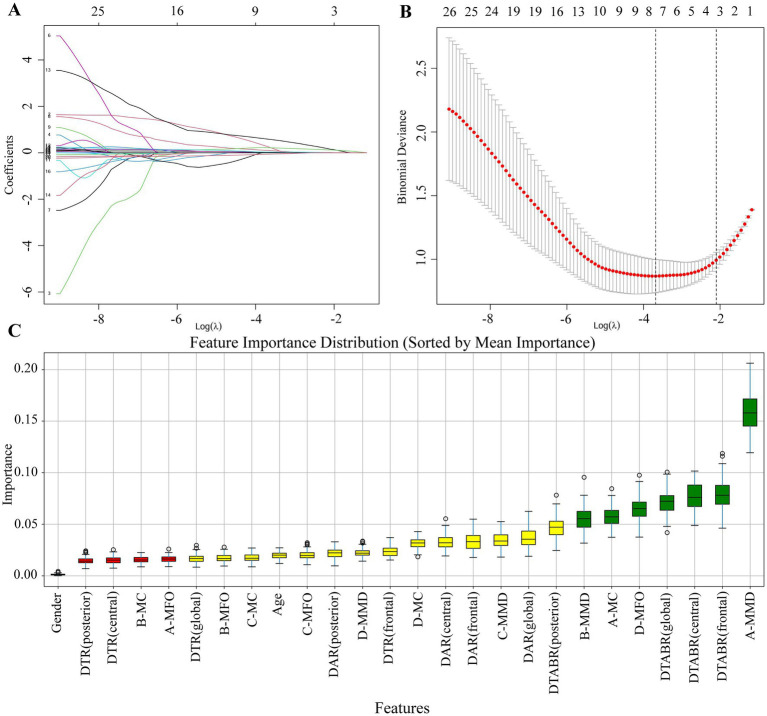
Feature selection process using LASSO and random forest. **(A)** Coefficient trajectories of candidate variables plotted against log(*λ*). Each colored line represents one variable, showing how coefficients shrink as λ increases. **(B)** Ten-fold cross-validation curve illustrating binomial deviance versus log(λ), with red dots indicating deviance values and error bars representing standard error. Seven variables were retained at the λ with minimum deviance, while four remained at the 1-SE criterion, achieving a balance between model performance and simplicity. **(C)** Each bar represents the importance score of a variable calculated by the random forest classifier. Variables shown in green were retained as important predictors, while those in yellow and red were excluded. A total of seven features were ultimately selected for further model development and analysis.

To identify EEG-based predictors of PSCI, a total of 26 candidate variables were initially considered, including demographic characteristics (age, sex) and 24 EEG-derived parameters. Feature selection was first performed using the least absolute shrinkage and selection operator (LASSO) regression. As shown in Figures 3A,B, seven variables were retained at the wavelength (*λ*) corresponding to the minimum deviance. Using the 1-Standard Error (1-SE) criterion, a more streamlined set of four variables—DTABR (global), DTABR (central), A-MMD, and A-MC—was chosen. This selection balances model simplicity with predictive accuracy.

To refine feature selection, a random forest algorithm was applied to rank the importance of all 26 candidate variables. As shown in Figure 3C, the top seven predictors—A-MMD, DTABR (frontal), DTABR (central), DTABR (global), D-MFO, A-MC, and B-MMD—were identified based on their high importance scores (green bars) and retained for model development. Additionally, the Boruta algorithm was employed to refine selection by identifying 13 features as confirmed important: DAR (global), DAR (frontal), DAR (central), DTABR (global), DTABR (frontal), DTABR (central), DTABR (posterior), A-MMD, A-MC, B-MMD, C-MMD, D-MFO, and D-MC. The intersection of features identified by LASSO, random forest, and Boruta resulted in seven final predictors used for subsequent model construction: A-MMD, DTABR (frontal), DTABR (central), DTABR (global), D-MFO, B-MMD, and A-MC. This consensus approach prioritizes features consistently deemed important, thereby minimizing the risk of selection bias associated with any single method.

### 3.3 Performance comparison of five machine learning models

Table 2 presents a comparative analysis of five machine learning (ML) models used for predicting PSCI: Random Forest, SVM, XGBoost, Logistic Regression, and Decision Tree. Among these, Random Forest achieved the best overall performance, with the highest accuracy (0.83) and F1 score (0.83), and a robust AUC of 0.91, indicating strong discrimination ability. To provide statistical certainty for the final model, we additionally report bootstrapped 95% confidence intervals for its key metrics: AUC = 0.91 (95% CI: 0.82–0.98) and accuracy = 0.83 (95% CI: 0.72–0.92). SVM showed the highest AUC (0.94) and specificity (0.92), suggesting excellent capability in identifying non-PSCI cases, although its sensitivity (0.71) was relatively lower than Random Forest, XGBoost, and Decision Tree. XGBoost achieved moderate performance with a balanced sensitivity (0.75) and specificity (0.88), but its Brier score (0.18) was higher than others, indicating slightly poorer calibration. Logistic Regression demonstrated competitive AUC (0.93) and high specificity (0.92), but relatively lower sensitivity (0.68), reflecting its limitation in detecting positive PSCI cases. Decision Tree, while having the lowest AUC (0.84), maintained decent overall performance with an accuracy of 0.79 and balanced sensitivity and specificity (both 0.75–0.84).

**Table 2:** Comparison of the characteristics of five ML models for predicting PSCI.

Model	Accuracy	AUC	Brier score	Sensitivity	Specificity	PPV	NPV	F1 score
Random forest	0.83	0.91	0.12	0.79	0.88	0.85	0.79	0.83
SVM	0.81	0.94	0.13	0.71	0.92	0.87	0.74	0.8
XGBoost	0.81	0.87	0.18	0.75	0.88	0.84	0.76	0.81
Logistic regression	0.79	0.93	0.13	0.68	0.92	0.86	0.72	0.78
Decision tree	0.79	0.84	0.17	0.75	0.84	0.81	0.75	0.79

In addition to test set performance, 10-fold cross-validation was conducted to evaluate model robustness. The mean AUCs were 0.91 ± 0.09 for Random Forest, 0.89 ± 0.12 for SVM, 0.88 ± 0.08 for XGBoost, 0.89 ± 0.09 for Logistic Regression, and 0.81 ± 0.07 for Decision Tree. These results were generally consistent with the AUCs obtained from the test set, indicating stable model performance. Minor differences between cross-validation and test set AUCs may be attributed to variations in data partitioning, sampling variability, and the sensitivity of specific models to different data distributions.

Overall, Random Forest outperformed other models in terms of generalization and predictive balance, making it a suitable candidate for PSCI risk classification.

### 3.4 Model performance evaluation

**Figure 4:**
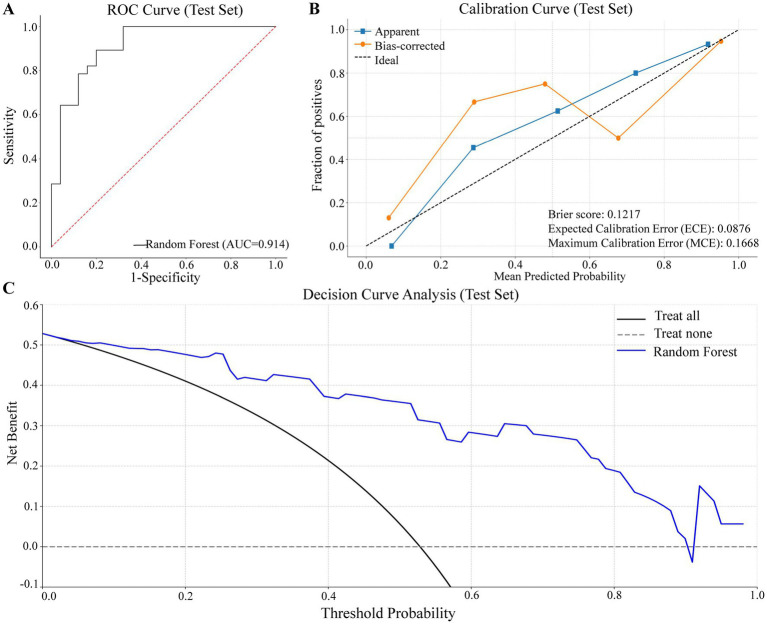
Internal validation performance of the prediction model. **(A)** ROC curve showing model discrimination (AUC = 0.91). **(B)** Calibration curve with apparent and bias-corrected lines. Brier score = 0.12, ECE = 0.09, MCE = 0.17. **(C)** Decision curve showing clinical net benefit across threshold probabilities.

As shown in Figure 4A, the model achieved an AUC of 0.91 in the test set, indicating excellent discriminative performance on new data. The calibration performance is summarized in Figure 4B. The Brier score was 0.12, demonstrating good overall accuracy of probabilistic predictions. The Expected Calibration Error (ECE) was 0.09, and the Maximum Calibration Error (MCE) was 0.17, reflecting acceptable agreement between predicted probabilities and observed outcomes. In the calibration plot, the apparent curve (blue) was closer to the 45-degree reference line, while the bias-corrected curve (orange), obtained via internal validation, showed slightly greater deviation, suggesting a conservative estimate of model performance.

As shown in Figure 4C, the decision curve analysis revealed that the Random Forest model (blue line) provided a higher net benefit than the “treat-all” and “treat-none” strategies across a wide range of threshold probabilities (approximately 0.05–0.70). The maximum net benefit was observed at thresholds around 0.1–0.2, highlighting the model’s potential clinical utility in supporting decision-making.

### 3.5 Visualization of feature importance using SHAP

**Figure 5:**
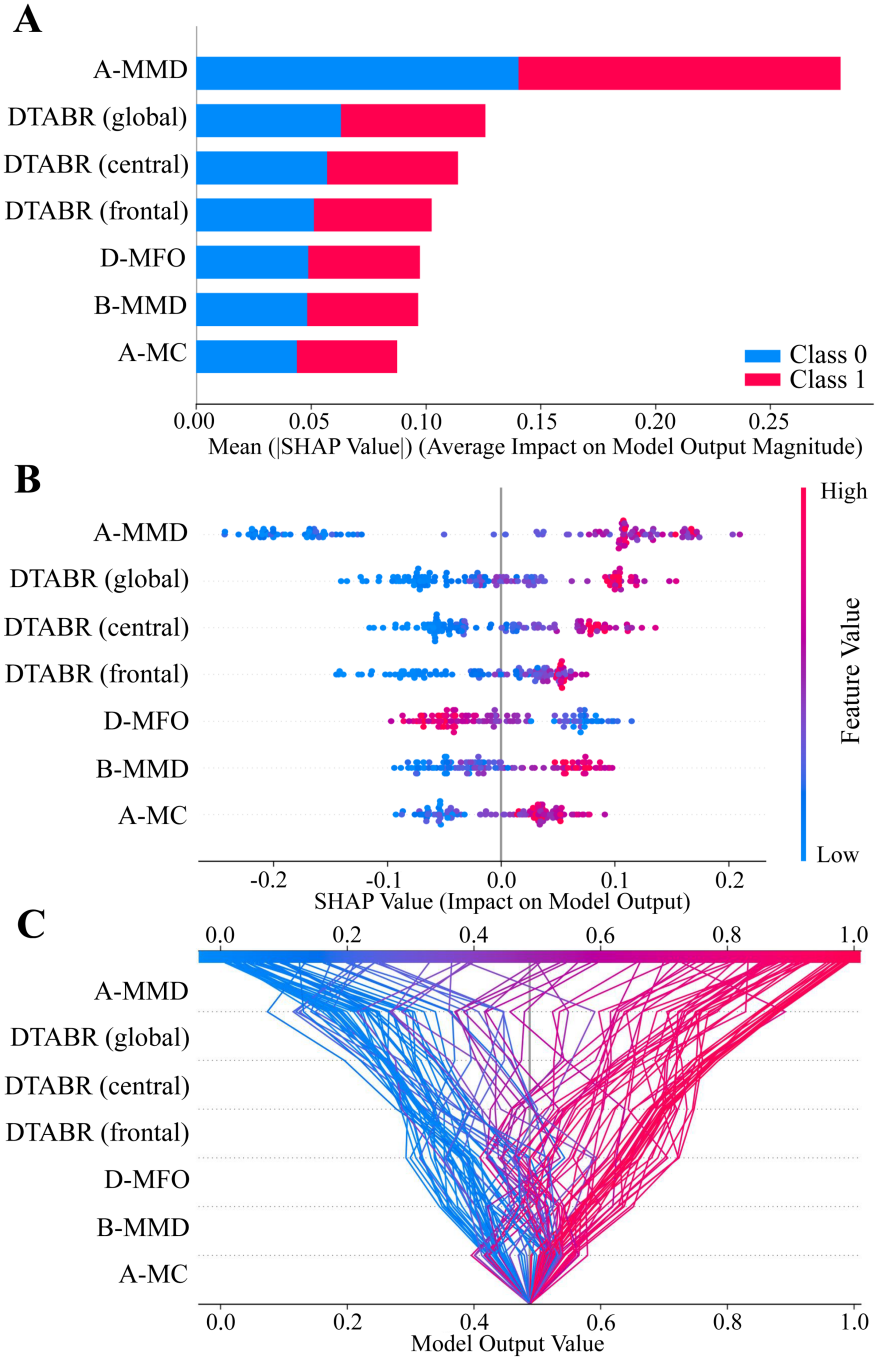
SHAP value summary plots of the best-performing model (Random Forest). **(A–C)** SHAP analysis illustrating feature contributions to model output. Each dot or line represents a SHAP value for an individual subject. The horizontal axis indicates the feature’s impact on the prediction. Feature importance is ranked by mean absolute SHAP values from top to bottom. Color represents original feature values (red = high, blue = low), and dot density reflects sample distribution.

SHAP analysis was used to interpret the Random Forest model for PSCI prediction. As shown in Figure 5A, A-MMD, DTABR (frontal, central, global), D-MFO, B-MMD, and A-MC were the top-ranked features based on their mean absolute SHAP values. Figure 5B illustrates the direction and magnitude of each feature’s impact on the model output. Red indicates high feature values, blue indicates low values. Higher values of A-MMD, DTABR, B-MMD, and A-MC were positively associated with PSCI risk, while higher D-MFO was linked to lower PSCI risk. Figure 5C presents a decision plot showing how individual features contribute to the model’s final prediction across subjects. The cumulative SHAP values demonstrate how key features influence the classification decision from baseline to final output.

### 3.6 Implementation of a web-based prediction tool for PSCI

**Figure 6:**
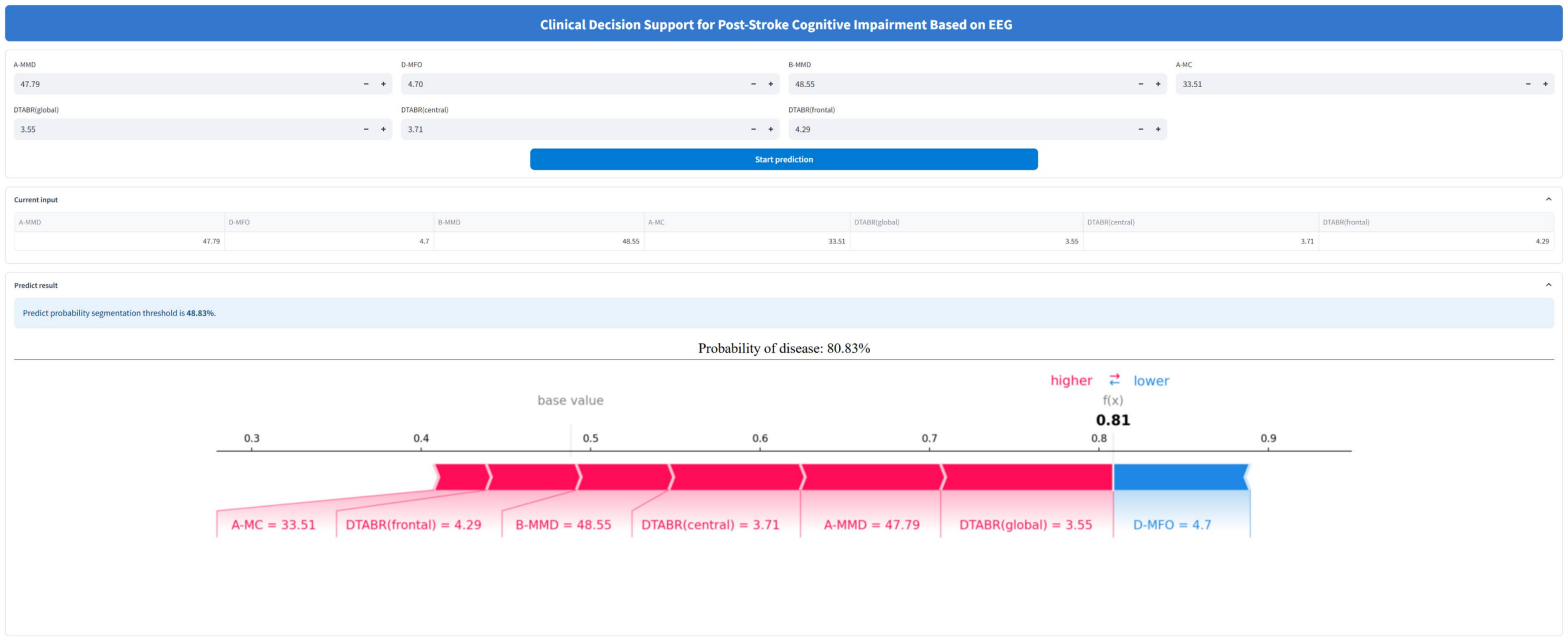
Application of a web-based clinical decision support system for PSCI using EEG signals. The final Random Forest model, based on seven key features, calculates and displays the probability of PSCI. After inputting the actual values of these seven features, the application automatically calculates and displays the probability of post-stroke cognitive impairment (PSCI). Meanwhile, the SHAP force plot for individual stroke patients visualizes the features contributing to the prediction: red features on the left increase the likelihood of PSCI, while blue features on the right reduce the likelihood and support a non-PSCI classification. The web-based prediction tool is available at: https://eeg-predict.streamlit.app/.

To facilitate clinical application, the final Random Forest model was deployed in a web-based clinical decision support system for PSCI prediction (Figure 6). This model integrates seven EEG-derived features and enables input of patient-specific data. Once the input is provided, the system automatically computes and displays the probability of PSCI. In the online platform, an individualized SHAP force plot is generated for each single patient rather than aggregated cohorts, providing a clear interpretation of feature contributions. In this plot, red bars on the left indicate variables that increase the likelihood of PSCI, while blue bars on the right represent those that reduce the risk. This individualized interpretability further enhances clinical utility by allowing transparent, patient-specific explanations. The tool is accessible at: https://eeg-predict.streamlit.app/. The optimal probability segmentation threshold for predicting PSCI is 48.83%.

### 3.7 External validation of the prediction model

The independent external validation cohort comprised 42 participants (20 PSCI, 11 PSN, 11 HC). The PSCI subgroup (*n* = 20) had a mean age of 62.4 ± 12.7 years, consisted of 12 males and 8 females, and included 4 hemorrhagic and 16 ischemic strokes with an equal distribution of left and right hemisphere lesions (10 each). The PSN subgroup (*n* = 11, all ischemic strokes) had a mean age of 65.5 ± 10.0 years, comprised 8 males and 3 females, and presented with 6 left-sided and 5 right-sided lesions. The HC group (*n* = 11) had a mean age of 67.5 ± 10.6 years and included 5 males and 6 females.

**Figure 7:**
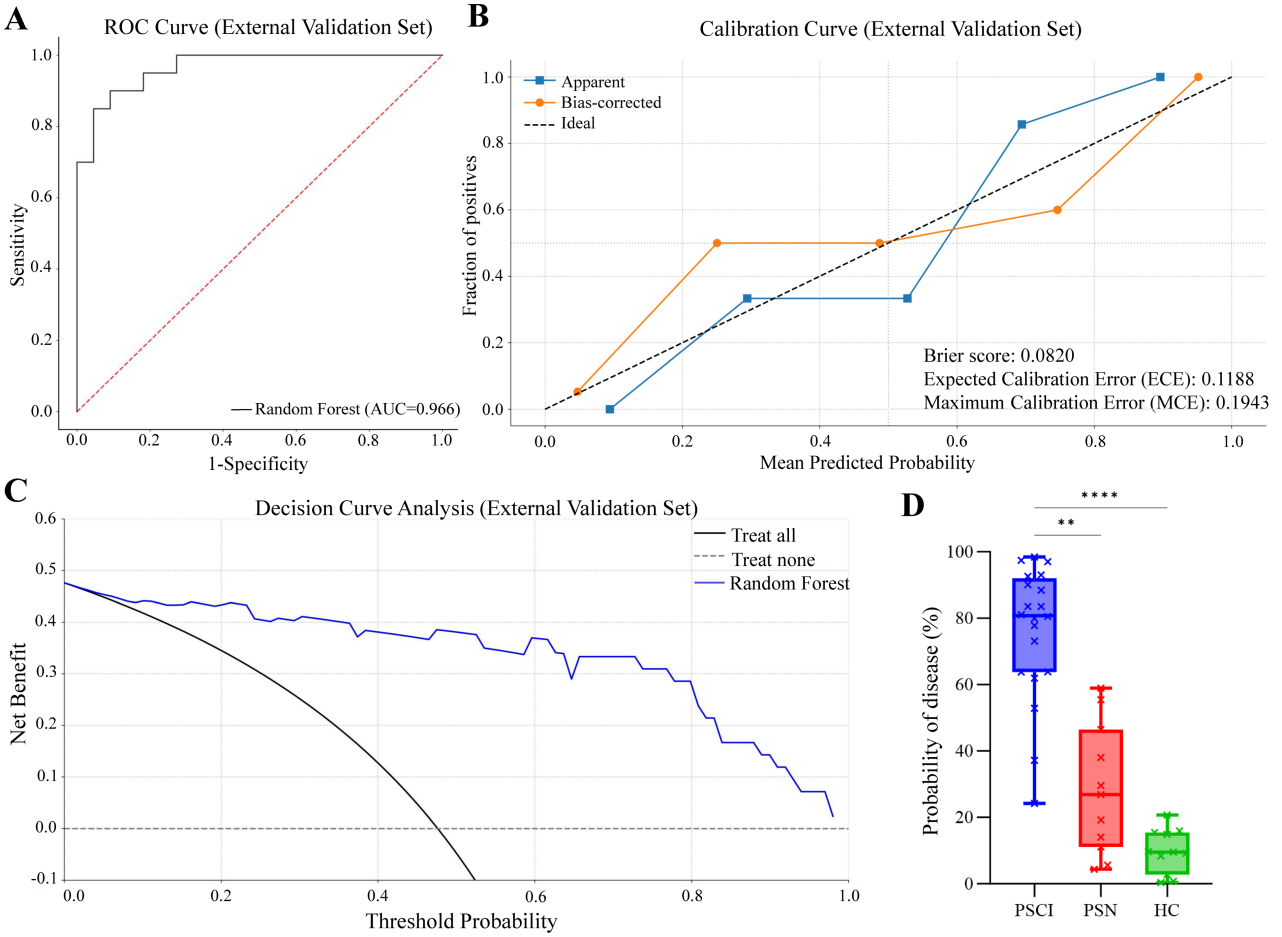
External validation performance of the prediction model. **(A)** ROC curve showing model discrimination (AUC = 0.97). **(B)** Calibration curve with apparent and bias-corrected lines (Brier score = 0.08). **(C)** Decision curve showing clinical net benefit across threshold probabilities. **(D)** Boxplots of individualized predicted probabilities for PSCI, PSN, and HC participants (**p < 0.01, ***p < 0.0001).

The final Random Forest model was applied to this external cohort for validation. As shown in Figures 7A–C, the model achieved an AUC of 0.97 and a Brier score of 0.08. The classification metrics were as follows: sensitivity = 0.90, specificity = 0.91, accuracy = 0.90, and F1 score = 0.90. Furthermore, decision curve analysis demonstrated a positive net clinical benefit across the full range of threshold probabilities (0.1–0.9).

Individualized predicted probabilities were visualized as boxplots for PSCI, PSN, and HC participants (Figure 7D). A Kruskal–Wallis test demonstrated significant differences among the three groups, and Dunn’s multiple comparisons test revealed significant differences between PSCI and HC, and PSCI and PSN, while differences between PSN and HC were not significant. These results collectively support the robustness and strong generalizability of the model on an independent external dataset.

## 4 Discussion

This study identified seven EEG-based features—A-MMD, DTABR (frontal, central, global), D-MFO, B-MMD, and A-MC—as important predictors of PSCI. The Random Forest model outperformed four comparative machine learning algorithms, achieving peak accuracy (0.83), F1 score (0.83), and excellent (AUC = 0.91) for PSCI prediction. The clinical utility of this method has been validated through several analyses, including ROC analysis, calibration curves, and DCA. According to established benchmarks for evaluating diagnostic tests, an AUC above 0.90—particularly when the lower bound of the 95% confidence interval exceeds 0.80—is considered indicative of excellent diagnostic accuracy (Çorbacıoğlu and Aksel, 2023).

The results showed good discriminative power and reliable probability calibration (Brier score = 0.12). There was also a significant net clinical benefit observed across threshold probabilities ranging from 0.05 to 0.7. Overall, this comprehensive validation supports the practical use of this method for the early identification of PSCI. Importantly, the clinical relevance of these findings is further reinforced by the neurophysiological basis of the selected features.

The seven selected features capture distinct post-stroke neurophysiological alterations, reflecting large-scale brain network dynamics changes that align with established mechanisms of cognitive dysfunction. Prolonged microstate durations in class A (auditory-temporal cortex) and class B (visuospatial-executive networks) suggest sustained activation in these distinct functional networks (Rubega et al., 2024). This pattern has been consistently associated with reduced neural flexibility and impaired efficiency in transitioning between cognitive states, key features of PSCI pathophysiology (Kong et al., 2025; Lian et al., 2021). Notably, these network-level dysfunctions are often accompanied by characteristic spectral changes in cortical activity. Similarly, elevated DTABR values in frontal, central, and global regions, representing greater slow-wave dominance over alpha–beta activity, suggest a shift toward cortical hypoactivation, which is strongly associated with reduced processing capacity in PSCI patients (Petrovic et al., 2017; Xu et al., 2024). Abnormal elevations in DTABR may therefore indicate the need for rehabilitation strategies aimed at improving processing capacity. Further, Increased mean coverage of microstate A (A-MC) observed in PSCI patients in our study is consistent with findings from Musaeus et al. (2019), who reported elevated A-MC in MCI and AD populations (Musaeus et al., 2019). Because microstate A is linked to temporal lobe activity—an area frequently affected in cognitive disorders—increased coverage of this microstate may indicate reduced neural adaptability and less efficient transitions between functional brain states (Chen et al., 2020; Musaeus et al., 2020). Moreover, abnormal increases in microstate A–related indices may indicate the need for training targeting responding and detection of target stimuli, given its association with sensory-driven response readiness (Zanesco et al., 2020). This pattern suggests a shared network-level vulnerability between degenerative and vascular cognitive impairments, highlighting A-MC as a potential electrophysiological marker of early cognitive dysfunction (Musaeus et al., 2019). By contrast, D-MFO—representing the mean frequency of occurrence of microstate D—was found to be negatively associated with PSCI risk. A higher D-MFO indicates more frequent engagement of attention-related networks, such as the frontoparietal systems, which play critical roles in maintaining cognitive stability and are often disrupted in PSCI (Frontzkowski et al., 2024; Kong et al., 2025; Zanto and Gazzaley, 2013). Thus, reduced D-MFO may indicate a need for attention-control–oriented cognitive training (Zanesco et al., 2020). Collectively, these features not only enhance the interpretability of the machine learning model but also reinforce the neurophysiological validity of our findings, aligning well with existing literature on EEG microstates and cognitive network dysfunction after stroke (Hao et al., 2022; Rubega et al., 2024; Wang et al., 2022).

Building upon this neurophysiological coherence, further evidence from Supplementary material 1 strengthens the validity of our findings. Comparisons of baseline characteristics and EEG parameters among PSCI, PSN, and HC groups revealed that most differences were statistically significant for PSCI vs. PSN and PSCI vs. HC (*p* < 0.05), whereas PSN and HC showed largely comparable profiles (*p* > 0.05). To further validate the clinical specificity of the final 7-feature set, we conducted pairwise discrimination analyses across the three subgroups (PSCI, PSN, and HC) (Supplementary material 3). The selected features demonstrated high sensitivity to cognitive impairment, specifically, distinguishing PSCI from PSN with an AUC of 0.92 (mean AUC = 0.83 ± 0.12) and PSCI from HC with an AUC of 0.99 (mean AUC = 0.96 ± 0.04). The lower discrimination accuracy observed between PSN and HC groups (AUC = 0.71) indicates that the selected biomarkers, especially the regional DTABR indices, reflect cognitive dysfunction rather than general post-stroke pathological changes. This indicates that despite potential statistical collinearity, the inclusion of regional indicators is clinically necessary to capture the subtle neurophysiological deviations unique to the cognitively impaired brain.

This phenomenon aligns closely with prior EEG literature. PSN individuals, often representing patients with milder stroke severity, typically exhibit resting-state EEG characteristics that more closely resemble healthy controls rather than cognitively impaired stroke survivors (Finnigan and van Putten, 2013). The reduced separability between PSN and HC therefore suggests that the EEG features identified in this study are more specifically associated with cognitive impairment instead of nonspecific effects of stroke itself. Furthermore, consistent with our findings, previous studies have also reported robust EEG differences between healthy individuals and patients with varying degrees of PSCI (Xu et al., 2024), reinforcing the neurobiological plausibility of these markers for cognitive screening.

To contextually evaluate these performance metrics, it is instructive to compare our current multi-modal approach with existing literature. First, contrasted with our group’s previous work which relied solely on a single microstate indicator—achieving a sensitivity of 80% but a specificity of only 69.6% (Kong et al., 2025)—the present integration of microstates with power spectral ratios has maintained comparable sensitivity (0.79) while significantly elevating specificity to 0.88. Second, our study diverges fundamentally in clinical objective from recent network-based investigations. For instance, while Lee et al. (2023a) utilized graph theory metrics to predict continuous 3-month MoCA scores (a regression task), our model is designed as a binary clinical decision support tool to facilitate rapid early triage (Lee et al., 2023a). Third, in terms of predictive accuracy, our model outperforms several recent algorithms relying on non-electrophysiological data. Models based on clinical and neuroimaging variables, such as those incorporating NIHSS scores, white matter lesions, or laboratory biomarkers (Lee et al., 2023b; Zhang et al., 2024), generally report AUCs ranging from 0.79 to 0.86. Unlike these approaches, which often depend on parameters that may not be routinely available in bedside settings, our EEG-based random forest model achieved superior discrimination using non-invasive, accessible data. Finally, while other EEG studies like Hadiyoso et al. (2023) have reported high classification accuracy, they were limited to distinguishing PSCI from healthy controls. By successfully differentiating PSCI from the more clinically challenging PSN group, our model demonstrates greater robustness for real-world application (Hadiyoso et al., 2023).

To translate this robust discriminative capability into accessible clinical practice and bridge translational gaps, we deployed our finalized model as a proof-of-concept web-tool that clinicians can use to assess PSCI risk in real time. Importantly, we tested this interface on an independent cohort during external validation, where it achieved an AUC of 0.97 and a Brier score of 0.08. These results underscore the model’s stability and suggest that it captures reproducible neurophysiological signatures rather than overfitting to our original dataset. While larger multicenter studies are still required to confirm its generalizability and integration into clinical workflows, this web-based deployment provides a scalable and interpretable framework for standardized PSCI risk stratification, providing the transparency required for medical decision-making (Sutton et al., 2020).

This study has several limitations. First, its single-center origin and modest sample size may affect generalizability, though the model’s strong performance in independent external validation is reassuring. Despite the significant group differences in sex distribution, sex did not emerge as an influential factor. It was not selected by any feature selection method, in line with previous evidence that sex has limited impact on EEG-based metrics (Karton et al., 2018). Second, the effective EPV in the training set was 8.9, marginally below the conventional threshold of 10; however, the use of robust machine learning techniques and successful external validation mitigates concerns about overfitting. Third, the impact of specific structural lesion locations was not analyzed, and future studies integrating neuroimaging data are needed. Finally, while PSCI classification was based on a combination of neurologist diagnosis and MoCA score, the absence of a full multidomain neuropsychological test battery must be noted. Future validation against comprehensive diagnostic standards is warranted.

## 5 Conclusion

This study identified seven EEG-derived microstate and spectral power ratio features that contribute meaningfully to the early identification of PSCI. By integrating these features, an interpretable Random Forest model was constructed, which demonstrated promising diagnostic performance in both internal validation and clinical utility evaluation. The deployment of this model via a user-friendly web-based interface offers a practical tool to assist in timely, objective PSCI risk assessment. While the current findings provide a foundation for EEG-based predictive modeling in cognitive neurology, future studies involving larger cohorts, more multidomain neuropsychological tests, and the incorporation of lesion localization data are warranted to refine predictive accuracy and support more personalized post-stroke rehabilitation planning.

## Data Availability

The raw data supporting the conclusions of this article will be made available by the authors, without undue reservation.
