# Supplementary material for: Identification and validation of an interpretable EEG-based machine learning model for the diagnosis of post-stroke cognitive impairment
Source: Front Aging Neurosci. 2026 Jan 12;17:1700771. doi: 10.3389/fnagi.2025.1700771 (PMC12832639; doi:10.3389/fnagi.2025.1700771)
Supplement: Supplementary file 1 [file Data_Sheet_1.pdf]

Supplementary Material

1 Supplementary material 1:

Tables 1. Comparison of baseline characteristics and EEG parameters between post-stroke cognitive impairment (PSCI), post-stroke non-cognitive impairment (PSN), and healthy control (HC) groups (* p<0.05, ** p<0.01, ***p<0.001, ns means p>0.05).

	PSCI (n=87)	PSN (n=35)	HC (n=52)	PSCI vs. PSN<br>*p* value	PSCI vs. HC<br>*p* value	PSN vs. HC<br>*p* value
Age	64.00 (54.00, 71.00)	61.00 (53.00, 67.00)	66.00 (59.00, 71.75)	ns	ns	ns
Sex				ns	***	**
Male	64	29	25			
Female	23	6	27			
Stroke type				ns	NA	NA
Ischemic	50	21	—			
Hemorrhagic	37	14	—			
Lesion lateralization				ns	NA	NA
Left hemisphere	40	12	—			
Right hemisphere	45	23	—			
**MoCA Total score**	14 (11, 19)	26 (26, 26)	26 (26, 26)	***	***	ns
**Power Ratio**						
DAR (global)	1.92 (0.85, 3.26)	0.76 (0.4, 2)	0.73 (0.48, 1.12)	***	***	ns
DAR (frontal)	2.8 (1.41, 4.8)	1.14 (0.53, 2.75)	0.97 (0.61, 1.81)	***	***	ns
DAR (central)	2.04 (0.81, 3.78)	0.74 (0.44, 2.01)	0.8 (0.54, 1.24)	***	***	ns

DAR (posterior)	1.03 (0.44, 2.57)	0.46 (0.23, 1.27)	0.38 (0.28, 0.79)	**	***	ns
DTR (global)	2.33 (1.26, 3.71)	1.92 (1.38, 2.79)	2.09 (1.55, 2.93)	ns	ns	ns
DTR(frontal)	2.52 (1.47, 4.67)	2.27(1.40, 3.21)	2.24 (1.59, 3.10)	ns	ns	ns
DTR(central)	2.42 (1.27, 3.57)	1.90 (1.28, 2.84)	2.16 (1.60, 2.93)	ns	ns	ns
DTR(posterior)	1.78 (1.05, 2.62)	1.67 (1.11, 2.31)	2.04 (1.30, 2.63)	ns	ns	ns
DTABR(global)	1.99 (1.19, 3.46)	0.79 (0.57, 1.81)	0.68 (0.50, 1.16)	***	***	ns
DTABR(frontal)	2.64 (1.92, 4.27)	1.43 (0.69, 2.33)	0.92 (0.63, 1.52)	***	***	ns
DTABR(central)	2.06 (1.19, 3.56)	0.75 (0.61, 1.73)	0.73 (0.56, 1.13)	***	***	ns
DTABR(posterior)	1.42 (0.68, 2.56)	0.37 (0.53, 1.33)	0.44 (0.32, 0.89)	***	***	ns
**EEG microstates**						
A-MMD	48.78 (46.16, 52.99)	43.36 (41.25, 47.91)	40.66 (38.37, 43.18)	***	***	*
A-MFO	4.72 ± 0.92	4.90 ± 0.93	5.20 ± 1.12	ns	*	ns
A-MC	26.06 ± 5.10	22.77 ± 4.22	21.58 ± 4.34	**	***	ns
B-MMD	48.36 ± 5.82	44.11 ± 4.30	41.32 ± 4.12	***	***	*
B-MFO	4.55 ± 0.92	5.01 ± 0.91	5.25 ± 1.02	*	***	ns
B-MC	23.62 (21.21, 27.47)	23.94 (21.26, 26.38)	22.31 (18.42, 23.55)	ns	ns	ns
C-MMD	50.67 ± 6.10	47.56 ± 5.63	45.00 ± 4.42	*	***	ns
C-MFO	4.76 ± 1.04	5.35 ± 1.07	5.72 ± 1.20	*	***	ns
C-MC	28.39 (23.37, 32.94)	31.38 (26.75, 33.83)	30.12 (24.00, 33.44)	ns	ns	ns
D-MMD	44.91 ± 5.83	42.43 ± 4.50	40.57 ± 5.71	ns	***	ns
D-MFO	4.27 (3.46, 4.87)	4.66 (4.03, 5.22)	5.15 (4.34, 5.53)	ns	***	ns
D-MC	17.81 (14.32, 21.69)	20.14 (16.11, 23.82)	20.72 (18.09, 22.77)	ns	**	ns

2 Supplementary material 2:

Figure 1. Group-Averaged EEG Power Topographies Across Four Frequency Bands (Delta, Theta, Alpha, Beta) in PSCI and Non-PSCI.

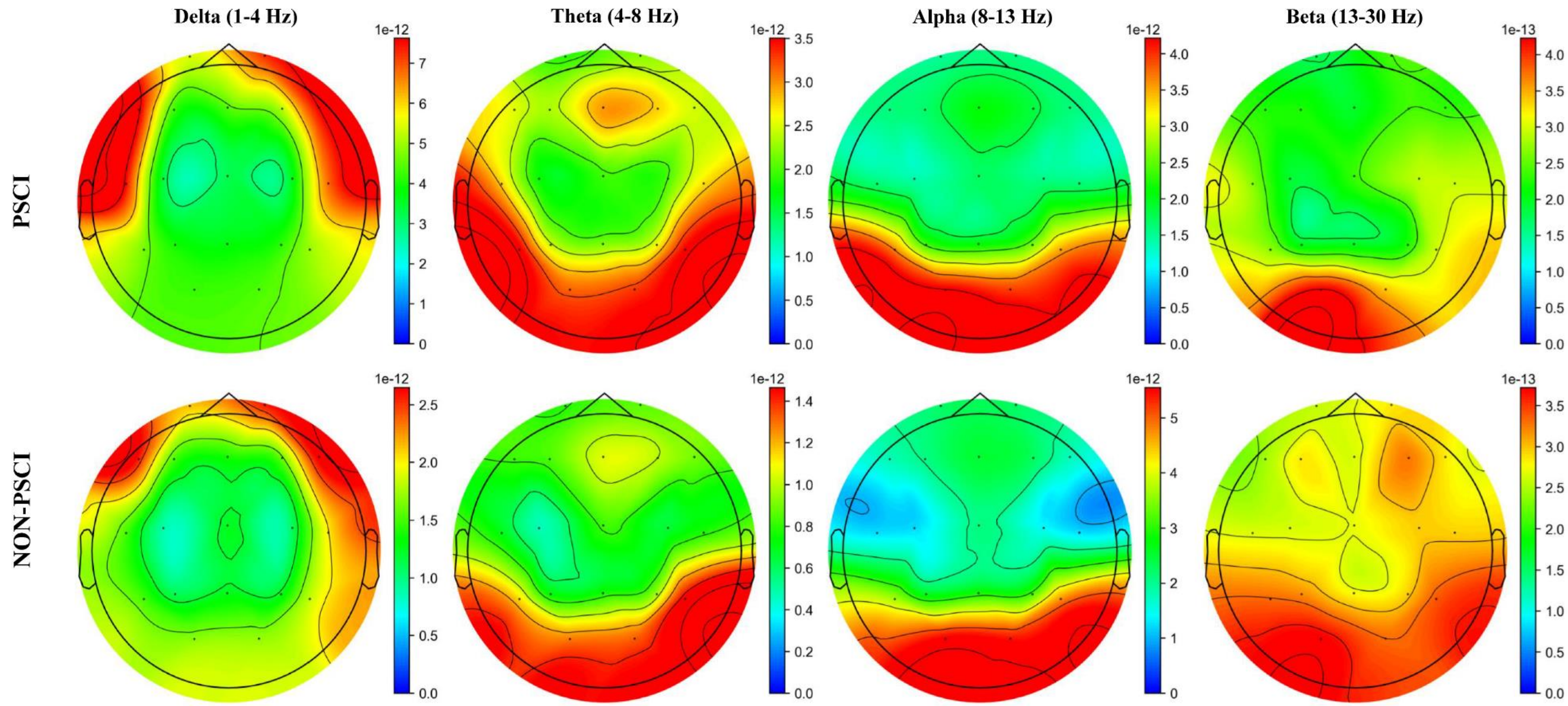

3 Supplementary material 3:

Table 2. Performance metrics of Random Forest models for the three pairwise group classifications.

Group	Accuracy	AUC	Mean AUC (K=10)	Brier Score	Sensitivity	Specificity	PPV	NPV	F1 Score
**PSCI vs. PSN**	0.85	0.92	0.83 ± 0.12	0.10	0.89	0.77	0.90	0.73	0.90

**PSCI vs. HC**	0.93	0.99	0.96 ± 0.04	0.06	0.89	1.00	0.99	0.84	0.94
**PSN vs. HC**	0.69	0.71	0.70 ± 0.21	0.24	0.23	1.00	0.89	0.66	0.37

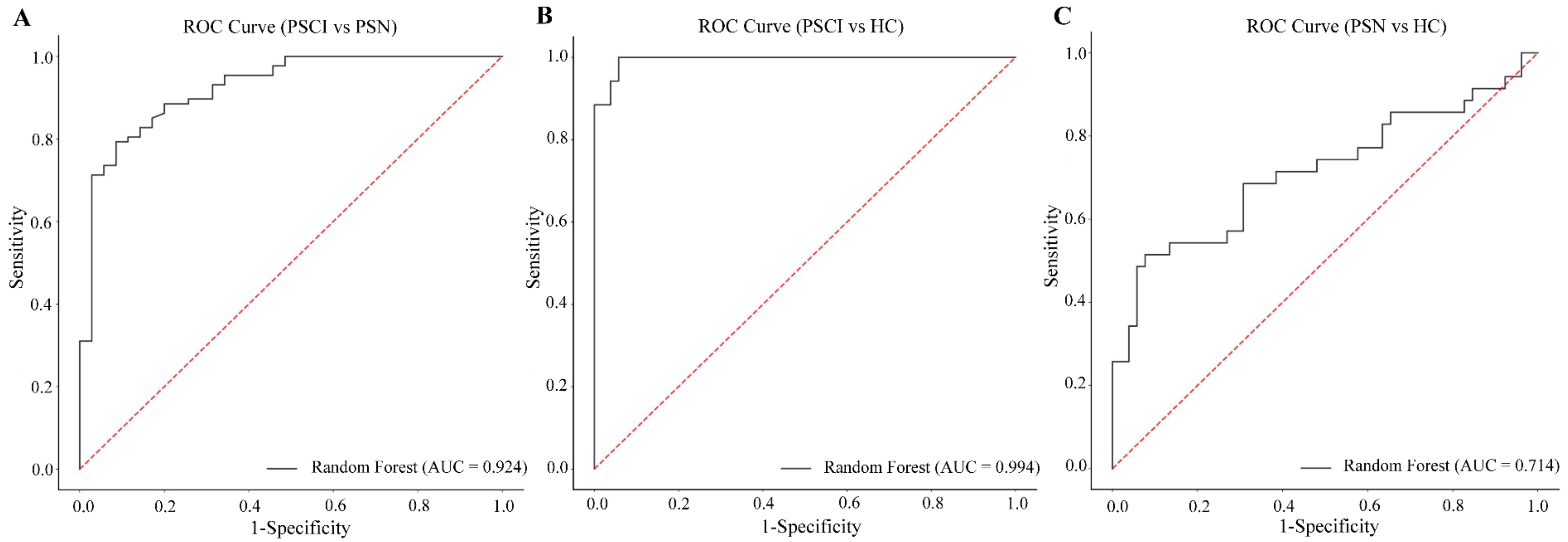

Figure 2. ROC curves of Random Forest models for the three pairwise comparisons: (A) PSCI vs. PSN, (B) PSCI vs. HC, and (C) PSN vs. HC, based on the seven key EEG features identified in the main analysis.

4 Supplementary material 4:

Disclaimer for the Web-Based Prediction Tool

This web-based prediction tool is intended solely for research purposes. It is not a medical device and must not be used for diagnostic decision-making, treatment planning, or patient management. The probability estimates and visual outputs generated by the platform—including individualized SHAP force plots—are derived from a machine learning model that remains under investigation.

The model has not undergone extensive multicenter external validation, regulatory review, or clinical certification. Its diagnostic performance may vary across populations, clinical settings, or EEG acquisition conditions. Users should be fully informed that the results

provided by this tool do not constitute medical advice and should not replace clinical judgment or formal cognitive assessment. By using this tool, users acknowledge and accept these limitations.
